# Molecular genetic characterisation of norovirus GII.17 strains circulating in South Korea in 2024

**DOI:** 10.2807/1560-7917.ES.2026.31.13.2500372

**Published:** 2026-04-02

**Authors:** Yunhee Jo, Minji Lee, Deog-Yong Lee, Myung-Guk Han, Sun-Whan Park

**Affiliations:** 1 Division of Viral Diseases, Department of Diagnosis and Analysis, Korea Disease Control and Prevention Agency, Cheongju, South Korea

**Keywords:** Norovirus, Kawasaki323, GII.17 variant, South Korea, Phylogenetic analysis, Mutation

## Abstract

**BACKGROUND:**

In South Korea, norovirus outbreaks have been predominantly attributed to the GII.4 genotype; however, since mid-2024, strains harbouring the GII.17 genotype are being detected more frequently, raising concerns about a potential shift in the dominant circulating strain and resulting public health implications.

**AIM:**

We aimed to analyse the nt sequences of the gene encoding the viral protein 1 (VP1), the major capsid protein, of the GII.17 norovirus strains detected in South Korea in 2024, compare them with the corresponding nt sequences of the GII.17 Kawasaki 2014 lineage strains and evaluate whether GII.17 has the potential to replace the long-dominant GII.4 genotype.

**METHODS:**

We obtained 11 complete VP1 sequences from stool specimens collected across six regions in South Korea (Busan, Daejeon, Gangwon-do, Gyeongsangbuk-do, Incheon, Jeollanam-do) in 2024 and compared them with the VP1 sequence of the reference strain (GII.17 Kawasaki 2014) to identify genetic changes.

**RESULTS:**

Key amino acid substitutions (R299P, N378D and K388R) were identified in the P2 domain of the VP1, which is a major antigenic site. Notably, N378D corresponds to D374 in the GII.4 genotype, which is a position involved in histo-blood group antigen (HBGA) binding, and K388R, adjacent to other antigenic sites, may alter the surface charge of the capsid.

**CONCLUSION:**

The data provide molecular evidence that the emerging GII.17 strain in South Korea may evade the host immune surveillance machinery and exhibit altered receptor binding, underscoring the public health threat posed by this strain and the need for enhanced genomic surveillance and effective vaccine development.

**Key public health message**

**What did you want to address in this study and why?**

This study investigated the emergence of the GII.17 norovirus genotype in South Korea, aiming to identify how it differs, genetically, from a reference strain. We wanted to understand if the genetic changes could explain why the GII.17 strain is emerging, which may have public health implications.

**What have we learnt from this study?**

This study revealed specific amino acid changes (R299P, N378D, K388R) in the emerging GII.17 norovirus genotype. These changes, particularly N378D and K388R, suggest this particular norovirus genotype might evade existing immunity, posing a possible public health risk.

**What are the implications of your findings for public health?**

The findings imply a potential rise in norovirus outbreaks due to the emerging GII.17 strain's possible ability to evade immunity and alter transmission.

## Introduction

Norovirus is one of the leading causes of acute gastroenteritis across all age groups. Among the norovirus genotypes, the GII.4 genotype has been dominant for over two decades [1]. The GII.17 Kawasaki 2014 variant temporarily replaced GII.4 as the dominant genotype in parts of East Asia between 2014 and 2016 [2-4]; however, the GII.4 genotype subsequently re-emerged as the dominant genotype globally [1,5].

Outside East Asia, since 2023–2024, GII.17 is also being increasingly detected in Europe and the United States (US), coinciding with a decline in the circulation of strains harbouring the GII.4 genotype [6,7].

The GII.17 Kawasaki strain - first identified in Kawasaki City, Japan - is different from the earlier GII.17 strains in that it harbours a viral protein 1 (VP1), the major capsid protein, which has two characteristic amino acid insertions in its P2 domain (the most surface-exposed region of VP1) [2]. The P2 domain mediates the binding of the VP1 to the host receptor proteins, i.e. histo-blood group antigens (HBGAs), and plays a critical role in immune evasion and determining the affinity of VP1 for these HBGAs [1,8-10].

The mutations identified in the GII.17 Kawasaki 2014 strain may have altered viral antigenicity and host cell binding properties, potentially enabling viruses with the GII.17 genotype to replace preexisting viruses with genotypes such as GII.4 [5]. Indeed, viruses with the GII.17 genotype are presumed to escape host immunity via antigenic mechanisms similar to those previously described for viruses with the GII.4 genotype [11]. Due to the high genetic diversity and rapid evolution of noroviruses, novel variants of a given genotype may emerge and replace existing genotypes [12], supporting the need to characterise their molecular features to aid vaccine development and preventive strategies. Despite ongoing efforts, the development of vaccines against noroviruses is hampered by various factors, including the extensive genetic diversity of the virus, the short-lived and genotype-specific immunity of the host following infection, the HBGA-dependent susceptibility of the host to infection and the lack of robust culture or animal models for studying the virus and testing the vaccines. These limitations indicate the need for detailed molecular analysis, such as that in the present study, to provide insights that can guide future vaccine design and evaluation [13,14].

During 2024, norovirus activity in South Korea followed the usual winter pattern and the frequency of GII.17 genotype detections began to increase from November within this seasonal trend and with GII.17 as the dominant genotype. Outbreaks were mainly reported in school, childcare and food-service settings, consistent with patterns observed in previous seasons.

Although viruses of the GII.17 genotype have been under continuous surveillance, comprehensive genome-level analyses of strains detected since the emergence of the GII.17 Kawasaki 2014 strain remain limited. In this study, we analysed the genetic sequences of the GII.17 norovirus strains circulating in South Korea in 2024 and compared them with those of the Kawasaki lineage to assess their mutational patterns. We also evaluated the potential of the GII.17 genotype to replace the long-dominant GII.4 genotype, emphasising the need for the continuous monitoring of variant trends.

## Methods

### Sample collection

During 2024, (January to December), outbreak cases were defined as cases associated with epidemiologically linked clusters, referring to two or more cases sharing a common exposure or transmission setting (e.g. the same facility, food source, or event), reported through the national outbreak surveillance system (Korean CaliciNet), the national laboratory surveillance system for norovirus operated by the Korea Disease Control and Prevention Agency (KDCA). During the same period, cases with sporadic acute gastroenteritis (non-outbreak cases, defined as sporadic cases identified through routine sentinel surveillance, without epidemiological linkage to a cluster) were identified through the Korean Enteric pathogen active surveillance Network (EnterNet-Korea) sentinel surveillance system operated by KDCA. In South Korea, regional Institutes of Health and Environment receive clinical specimens from collaborating hospitals and local public health centres as part of the national surveillance system. During 2024 (January to December), stool specimens from outbreak and non-outbreak cases were provided by 18 regional Institutes of Health and Environment across six regions in South Korea and submitted to KDCA for laboratory analysis. Viral RNA was extracted from the stool specimens and subsequently amplified by PCR for molecular characterisation. No personal identifiers were included, and only limited epidemiological information was available. For outbreak cases identified through the Korean CaliciNet, cases from all age groups were included. In contrast, for non-outbreak cases identified through the EnterNet-Korea sentinel surveillance system, age-related information was available only for children under five years of age.

Among samples collected throughout 2024, we focused on specimens collected between November and December 2024, when the frequency of GII.17 genotype detections began to increase, and selected 11 stool specimens that tested positive for norovirus by multiplex real-time RT-PCR and subsequent amplification of the capsid and RNA-dependent RNA polymerase genes. These corresponded to six outbreak-associated cases in the regions of Busan, Incheon and Jeollanam-do, and five non-outbreak cases in the regions of Daejeon, Gangwon-do, Gyeongsangbuk-do and Jeollanam-do.

### Laboratory investigations and phylogenetic analysis

Norovirus infection was diagnosed using a multiplex real-time reverse transcription PCR assay (PowerChek Norovirus GI/GII Multiplex Real-time PCR Kit, Kogene Biotech, Seoul, South Korea). Specimens were considered norovirus-positive if RNA corresponding to both the capsid and RNA-dependent RNA polymerase (RdRp) genes was successfully amplified using the HyQOne step RT-PCR premix kit (dye plus) (iMOD-001TD, SNC, Seoul, South Korea). Genotyping was conducted by sequencing a 570 bp fragment spanning the open reading frame (ORF)1-ORF2 junction using type-specific primers and sequences were assigned to genotypes using the Norovirus Genotyping Tool, as described previously [15].

A full-length sequence of the GII.17 VP1 gene (1,623-nt long) was used to conduct a phylogenetic analysis using Molecular Evolutionary Genetics Analysis Software (MEGA) version 11 (www.megasoftware.net). The analysis included a total of 36 sequences, 25 reference norovirus GII.17 sequences retrieved from GenBank, corresponding to GII.17 strains reported between 1976 and 2024, including the Kawasaki lineage strain (Hu/GII/JP/2014/Kawasaki323) and the 11 new sequences collected between November and December 2024 in South Korea as described above. A phylogenetic tree was constructed using the maximum-likelihood method and the reliability of the tree was assessed through 1,000 bootstrap replicates. The clade position of South Korean GII.17 strains was compared with that of the Kawasaki lineage strains to determine their genetic relationship.

### Amino acid sequence comparison

Amino acid sequences were deduced from the obtained nucleotide (nt) sequences and aligned with those of the reference GII.17 Kawasaki strain to identify mutation sites. Particular attention was paid to substitutions, insertions or deletions occurring in VP1 epitope regions. Amino acid positions were assigned based on the sequence of the reference strain (Kawasaki323; GenBank accession number AB983218). The biological significance of the observed mutations was interpreted based on previous literature.

## Results

### Epidemiological context

During November and December 2024, norovirus outbreaks were reported in several regions of South Korea accompanied by a marked increase in the proportion of the GII.17 genotype, rising from less than 1% in previous years to 32.6% from late 2024 to 2025. Outbreak settings, including the distribution of age groups and sex ratios, remained largely comparable to those observed in prior seasons. School and childcare facilities as well as food service establishments were most frequently reported as outbreak locations. Limited demographic information was available for the 11 GII.17-positive specimens analysed. Outbreak cases identified through the Korean CaliciNet included individuals from all age groups. In contrast, non-outbreak cases identified through the EnterNet-Korea sentinel surveillance system were restricted to children under 5 years of age. Age information was available only for a subset of cases. Consequently, no meaningful comparison could be made between patients infected with Kawasaki lineage strains and those infected with Cluster IV strains (GII.17 strains detected in South Korea in 2024).

### Phylogenetic analysis of the nucleotide sequences

**Figure 1:**
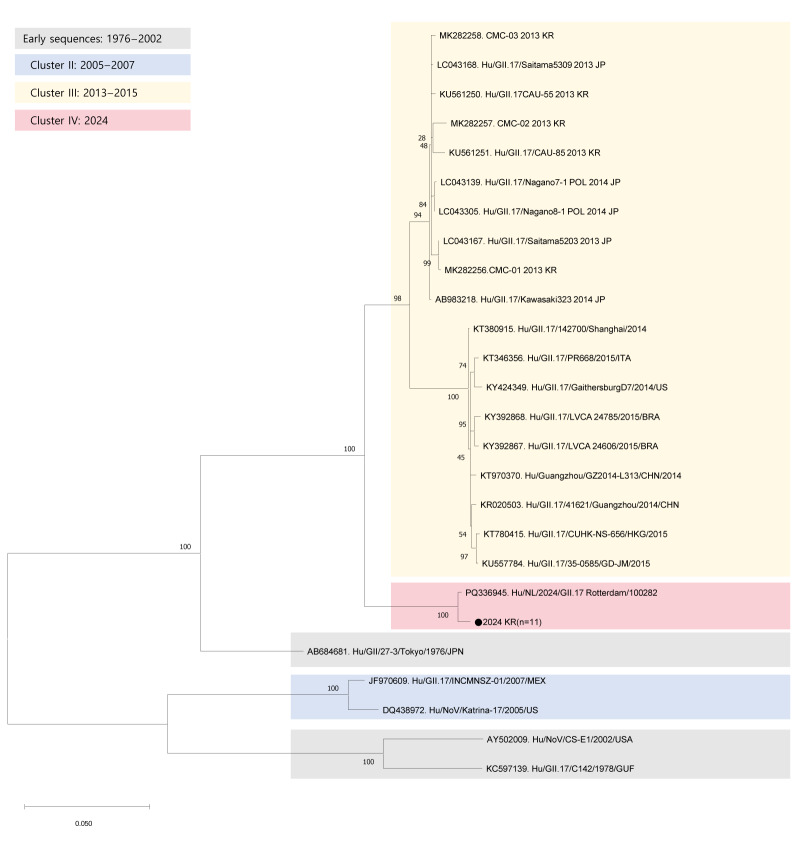
Maximum-likelihood method-based phylogenetic analysis of the nucleotide sequences of the gene encoding the viral protein 1 in GII.17 strains, since including the norovirus strains isolated from South Korea in 2024

Genotyping showed that while several norovirus genotypes were detected in South Korea during the study period, the majority of the sequences analysed in this study corresponded to the GII.17[P17] strain. The norovirus strains detected in South Korea in 2024 clustered closely with a strain isolated from the Netherlands in 2024 (Hu/NL/2024/GII.17/Rotterdam/00822), forming a lineage genetically distinct from the pandemic Kawasaki323 variant from 2014 (Figure 1). Basic metadata of the South Korean strains, including collection data and location, are summarised in Table 1.

The phylogenetic tree was constructed using the maximum-likelihood method, using the sequence of the full-length gene encoding the GII.17 VP1 protein; the strains used for gene amplification included 25 reference strains from GenBank and 11 isolates collected from South Korea in 2024 which is indicated by ●; KR, n = 11. Bootstrap values (1,000 replicates) above 70% are indicated at the nodes. The scale bar indicates the branch length corresponding to 0.050 substitutions per 100 nt sites. Four distinct clusters are marked by shaded boxes: (i) early sequences (1976–2002; grey), (ii) Cluster II (2005–2007; blue), (iii) Cluster III (2013–2015; yellow), and (iv) Cluster IV (2024; pink).

**Table 1:** Metadata of the GII.17 strains detected in South Korea in 2024, (n = 11)

Strain ID	Collection month	Location of strain detection	Outbreak context
KOR2024–01	Dec	Jeollanam-do	Outbreak
KOR2024–02	Dec	Jeollanam-do	Outbreak
KOR2024–03	Dec	Jeollanam-do	Outbreak
KOR2024–04	Dec	Jeollanam-do	Outbreak
KOR2024–05	Dec	Incheon	Outbreak
KOR2024–06	Nov	Jeollanam-do	Non-outbreak
KOR2024–07	Dec	Daejeon	Non-outbreak
KOR2024–08	Nov	Gangwon-do	Non-outbreak
KOR2024–09	Dec	Gangwon-do	Non-outbreak
KOR2024–10	Dec	Gyeongsangbuk-do	Non-outbreak
KOR2024–11	Dec	Busan	Outbreak

ID: identification.

Each strain is listed with its collection date, location, and outbreak context (outbreak or non-outbreak case). This table summarises the metadata of the South Korean strains to support the interpretation of the phylogenetic clustering shown in Figure 1.

Phylogenetic analysis showed that the three reference sequences obtained between 1976 and 2002 did not form a consistent cluster but rather were grouped into several distinct lineages. The three major clusters that emerged after 2005 were named Cluster II (2005–2007), Cluster III (2013–2015) and Cluster IV (2024, which included the 11 GII.17 strains detected in South Korea in November–December 2024). Cluster IV was distinct from Cluster III, which included the GII.17 Kawasaki 323 (2014) reference strain (Figure 1).

### Amino acid substitution analysis of viral protein 1, the major capsid protein

Sequence alignment of the VP1 (at the amino-acid level) of the GII.17 strains detected in South Korea in 2024 with those of the Kawasaki lineage strains revealed numerous amino acid substitutions and several insertions. Most of the mutations were located in the P2 domain, which is a major antigenic site of the VP1 [16,17]; the sequence of the P2 domain was analysed to identify substitution mutations that may influence antigenicity and host interactions (Table 2).

**Table 2:** Key amino acid substitution positions in the viral protein 1 of the GII.17 norovirus strains detected in South Korea in 2024

Strain number	Position^a^	Substitution (Ref → 2024 strain)	Region/domain	Novelty
1	298	H → N	P2 domain	Reported
2	299	R → P	P2 domain	Reported
3	336	V → M	P2 domain	Reported
4	352	A → S	P2 domain	Novel
5	363	Q → R	P2 domain	Reported
6	374	R → K	P2 domain	Reported
7	378	N → D	P2 domain	Reported
8	388	K → R	P2 domain	Reported
9	399	D → N	P2 domain	Novel
10	404	H → Q	P2 domain	Novel
11	417	L → V	P2 domain	Reported

A: alanine; D: aspartic acid; H: histidine; K: lysine; L: leucine; M: methionine; N: asparagine; P: proline; Q: glutamine; R: arginine; S: serine; V: valine.

^a^ Amino acid substitution positions assigned based on the numbering used in the sequence of the Kawasaki323 reference strain (GenBank accession number AB983218).

The table lists amino acid substitutions at variable positions within the P2 domain of the VP1 among the GII.17 strains detected in South Korea. Only sites with at least one substitution are included.

The strains detected in South Korea in 2024 exhibited several amino acid substitutions (comparator, the Kawasaki323 strain), most of which were located in the P2 domain. These included new mutations as well as old mutations; substitutions at positions 352, 399, and 404 were absent in the 2014 Kawasaki strain but appeared newly in strains detected in South Korea and Rotterdam in 2024 (new mutations); the remaining substitution mutations had already been reported in GII.17 strains circulating after 2014 (old mutations).

Notably, all amino acid substitutions identified in the norovirus strains detected in South Korea in 2024 were located within the P2 domain of the VP1, indicating potential antigenic drift.

**Figure 2:**
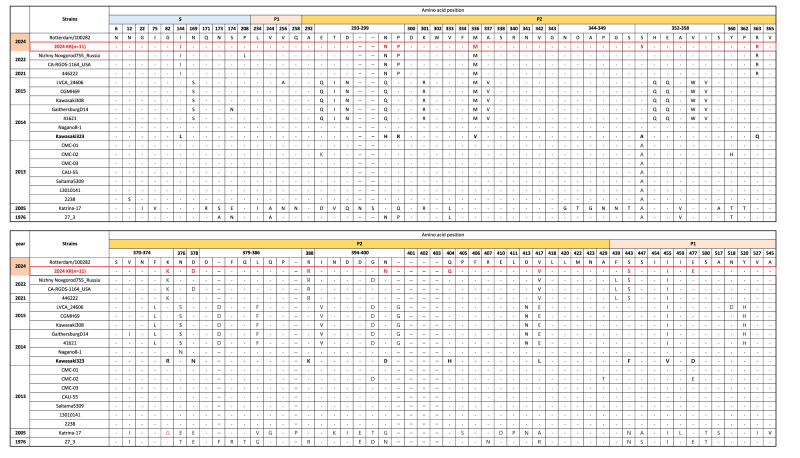
Comparative amino acid alignment of the GII.17 viral protein 1 P2 domain in the reference strain (Kawasaki323) and the norovirus strains detected in South Korea in 2024

One key mutation, N378D in the VP1 protein of the GII.17 strains, corresponds to the D374 residue in the VP1 of the GII.4 strains, which is known to be part of the HBGA-binding interface [18] (Figure 2). These substitutions were consistently observed across all the 11 strains isolated and analysed in this study, suggesting potential implications in immune recognition and vaccine design. This mutation pattern is consistent with changes observed in GII.17 variants that emerged after 2014, which acquired more than 20 substitution mutations in the P2 domain of the VP1 (comparator, pre-2024 strains) [19], suggesting that the antigenic profile of these viruses has evolved from the early Kawasaki lineage. These findings imply that the GII.17 variants may have undergone evolution to escape preexisting host immunity (Figure 2).

The schematic alignment shows amino acid substitutions in the P2 domain of the GII.17 viral protein 1 (VP1) of norovirus strains detected in South Korea (2014–2024) relative to that in the reference strain, i.e. Kawasaki323 (2014, JP; GenBank accession number AB983218). Additionally, several recent VP1 sequences, including OP805362 (Romania, 2021, partial VP1), ON854114 (Russia, 2022, partial VP1) and OP689689 (US, 2022, complete genome) have also been used for comparison. The partial VP1 sequences (OP805362 and ON854114) were not included in the phylogenetic tree (Figure 1), which was reconstructed from full-length VP1, while OP689689 was retained here for substitution comparison but not incorporated into the tree analysis. Only variable sites are shown. En dashes (–) indicate amino acids identical to the ones in the reference strain. The strains detected in 2024 are highlighted using a red box; the findings indicate shared substitution mutations distinct from those in the Kawasaki323 strain. Key substitutions, such as R299P, Q363R and K388R may indicate antigenic drift. Residue positions correspond to the amino acid numbering used in the Kawasaki323 strain.

## Discussion

This study confirmed that GII.17 noroviruses circulating in South Korea in 2024 exhibited high genetic similarity to the Kawasaki 2014 lineage strains while also harbouring distinct amino acid mutations.

This finding is indicative of the continuous evolution of GII.17 norovirus strains and the potential emergence of novel variants. The emergence of Cluster IV may be of epidemiological importance, as its diversification could facilitate broader community spread, underscoring the importance of continued molecular surveillance in South Korea.

Substitution mutations such as R299P and K388R may alter the charge distribution on the surface of the capsid, potentially affecting receptor binding or immune recognition. Notably, several substitutions identified in the GII.17 strains detected in South Korea in 2024 were located at positions homologous to the antigenic sites of GII.4 strains, at which amino acid changes have been implicated in immune escape and antigenic drift [8,9]. These findings suggest that the emergence of the GII.17 genotype occurred in parallel with the usual seasonal upsurge rather than being linked to a specific demographic shift or outbreak setting. Crucially, multiple substitutions and insertions were identified in the P2 domain of the capsid protein VP1, which is known to be critical for binding to human HBGAs and for recognition by neutralising antibodies. Even minor changes in this domain can considerably impact viral antigenicity and immune-escape potential [10].

Matsushima et al. [11] reported concentrated amino acid substitutions near the HBGA-binding interface in Japanese GII.P17-GII.17 strains. These changes were similar to the immune-escape patterns commonly observed in GII.4 strains. Given that most of the current norovirus vaccine candidates are based on GII.4-derived antigens, antigenic drift in GII.17 could potentially compromise cross-protection and vaccine efficacy. Similarly, the antigenic changes identified in our study may also contribute to immune evasion and may potentially influence viral transmissibility, possibly explaining the rapid emergence and predominance of the GII.17 variants both in South Korea and around the world. Recent surveillance reports on norovirus activity further reinforce this trend, documenting increased circulation of GII.17 strains in Europe and the US during the 2023/24 season [6]; and a report of a multi-province outbreak linked to a novel GII.17 lineage strain in Argentina in 2024 [18]. These findings highlight the global implications of GII.17 emergence and support the need for continued molecular surveillance at global level.

This study had some limitations, including the small number of analysed samples, limited availability of demographic information and focus on VP1 sequences only without analysis of other genomic regions. Moreover, the study period was restricted to a single year (2024), which may not fully capture longer-term evolutionary trends. These limitations should be considered when interpreting the findings of the study. In addition, detailed epidemiological information, including travel history, was not available for the analysed cases.

Although functional assays such as neutralisation tests were not performed, our findings raise the possibility that antigenic changes such as those reported here could potentially compromise cross-protection and effectiveness of future norovirus vaccines. Furthermore, nt sequences identical to those analysed in this study have also been detected in recent foetal norovirus infection cases in South Korea (data not shown).

## Conclusions

These findings underscore the importance of continued genomic surveillance and in-depth pathogenicity studies to better understand the public health risks associated with the emerging GII.17 norovirus variants. Continued monitoring of circulating strains will be important to detect further genetic changes and to assess their potential impact on public health.

## Data Availability

The nucleotide sequence data generated in this study have been deposited in GenBank under accession number PX470709.
